# Supplementary figures and images for: Gene expression signatures between Limia perugiae (Poeciliidae) populations from freshwater and hypersaline habitats, with comparisons to other teleosts
Source: PLoS One. 2024 Dec 5;19(12):e0315014. doi: 10.1371/journal.pone.0315014 (PMC11620662; doi:10.1371/journal.pone.0315014)

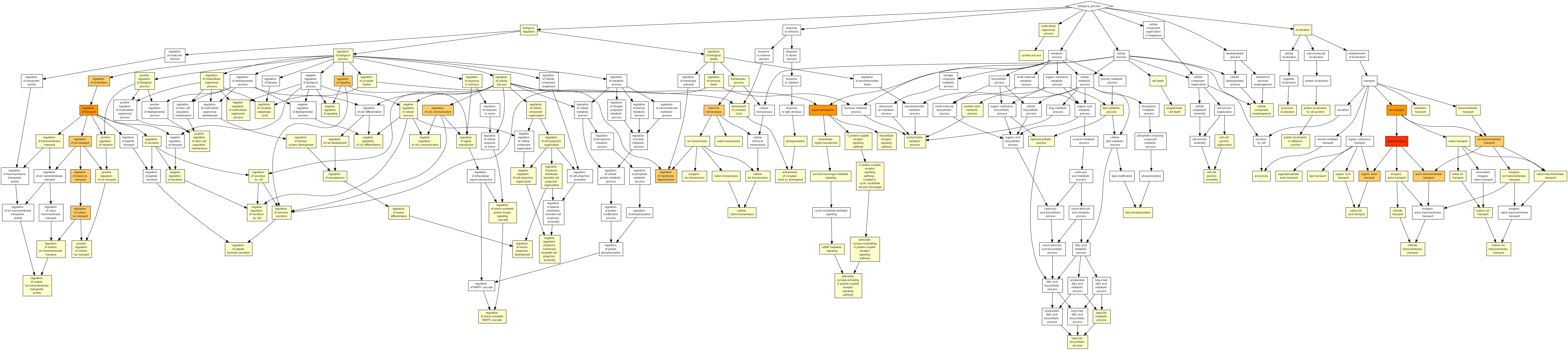

Supplement: S1 Fig — (PNG) [file pone.0315014.s001.png]

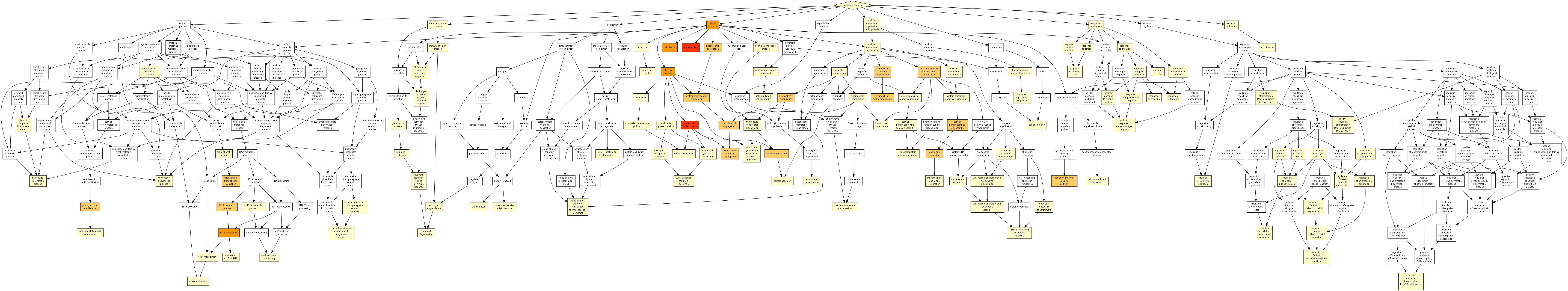

Supplement: S2 Fig — (PNG) [file pone.0315014.s002.png]

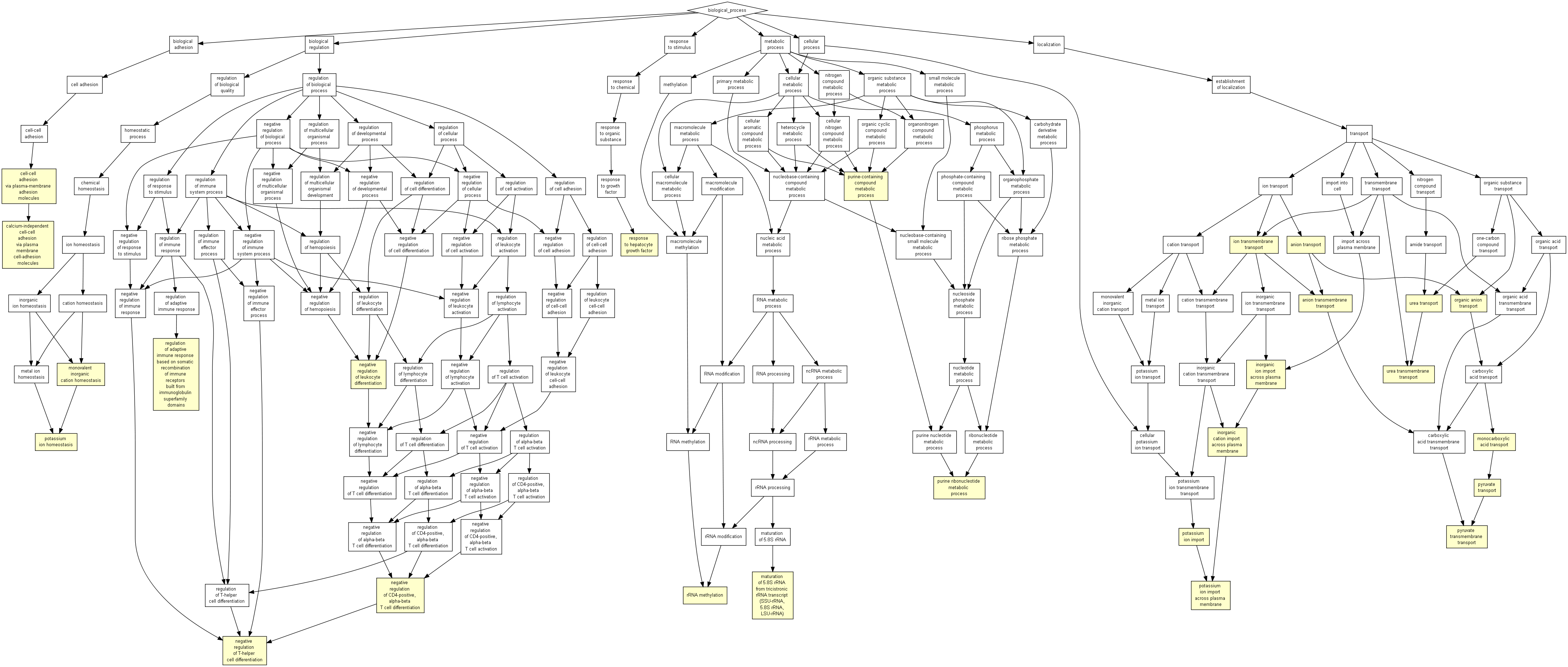

Supplement: S3 Fig — (PNG) [file pone.0315014.s003.png]
