# Supplementary material for: Gene expression signatures between Limia perugiae (Poeciliidae) populations from freshwater and hypersaline habitats, with comparisons to other teleosts
Source: PLoS One. 2024 Dec 5;19(12):e0315014. doi: 10.1371/journal.pone.0315014 (PMC11620662; doi:10.1371/journal.pone.0315014)
Supplement: Attachment — Submitted filename: 1130205_Reviewer Comments for Manuscript ID PONE-D-23-43144.docx [file pone.0315014.s005.docx]

Subject: Comprehensive Reviewer Comments for Manuscript ID PONE-D-23-43144

I have completed a thorough review of the manuscript titled "Gene expression signatures of salinity transitions in *Limia perugiae* (Poeciliidae), with comparisons to other teleosts" by Elizabeth J. Wilson et al., submitted for consideration in PLoS One (Manuscript ID: PONE-D-23-43144). Overall, the study provides valuable insights into the gene expression patterns associated with salinity transitions in *Limia perugiae*. However, I have identified several points that require clarification or further attention:

Title Clarification:

Given the study involves *Limia perugiae* populations from a hypersaline lagoon and a freshwater stream, rather than transitions between seawater and freshwater, the title may benefit from clarification. I recommend considering a title such as "Gene expression signatures in *Limia perugiae* populations from hypersaline and freshwater habitats" to accurately reflect the environmental context.

Salinity Percentage in Hypersaline Lagoon:

The authors are encouraged to provide information on the salinity percentage in the hypersaline lagoon (Laguna Oviedo: 17.801° N, 71.363° W). Clarifying the salinity range or percentage in the hypersaline lagoon is crucial for a comprehensive understanding of the study system.

SRA Accessions in Table 1:

In Table 1, the term "Pending" under the SRA Accessions column needs to be addressed. I recommend that the authors provide the specific SRA Accessions or update the information to ensure the reproducibility and accessibility of their RNA-seq data.

Explanation of Abbreviations in Figure 2(A):

In Figure 2(A), clarification is needed for the abbreviations "DC" and "MD" associated with the color bars below the plot. The authors should explicitly state the meanings of these abbreviations, aiding readers in the accurate interpretation of the data presented.

Confirmation of Transcriptomic Data through Real-time qPCR:

The manuscript mentions that confirmation of the transcriptomic data through real-time quantitative PCR (qPCR) is pending. I suggest the authors emphasize the importance of validating their RNA-seq results using qPCR and discuss how this confirmation would strengthen the reliability of their findings.

In addition to these specific points, I commend the authors for their comprehensive analysis of gene expression patterns in *Limia perugiae* and the comparison with other teleosts. The study contributes significantly to our understanding of salinity transitions in fish. Addressing the mentioned concerns will further enhance the clarity, reproducibility, and overall quality of the manuscript.
